# Multiple Conserved Heteroplasmic Sites in tRNA Genes in the Mitochondrial Genomes of Terrestrial Isopods (Oniscidea)

**DOI:** 10.1534/g3.115.018283

**Published:** 2015-04-24

**Authors:** Christopher H. Chandler, Myriam Badawi, Bouziane Moumen, Pierre Grève, Richard Cordaux

**Affiliations:** * Department of Biological Sciences, SUNY Oswego, Oswego, New York 13126; † Université de Poitiers, UMR CNRS 7267 Ecologie et Biologie des Interactions, Equipe Ecologie Evolution Symbiose, TSA 51106, 86073 Poitiers Cedex 9, France

**Keywords:** heteroplasmy, dual tRNA, Oniscidea, mitogenome

## Abstract

Mitochondrial genome structure and organization are relatively conserved among metazoans. However, in many isopods, especially the terrestrial isopods (Oniscidea), the mitochondrial genome consists of both ∼14-kb linear monomers and ∼28-kb circular dimers. This unusual organization is associated with an ancient and conserved constitutive heteroplasmic site. This heteroplasmy affects the anticodon of a tRNA gene, allowing this single locus to function as a “dual” tRNA gene for two different amino acids. Here, we further explore the evolution of these unusual mitochondrial genomes by assembling complete mitochondrial sequences for two additional Oniscidean species, *Trachelipus rathkei* and *Cylisticus convexus*. Strikingly, we find evidence of two additional heteroplasmic sites that also alter tRNA anticodons, creating additional dual tRNA genes, and that are conserved across both species. These results suggest that the unique linear/circular organization of isopods’ mitochondrial genomes may facilitate the evolution of stable mitochondrial heteroplasmies, and, conversely, once such heteroplasmies have evolved, they constrain the multimeric structure of the mitochondrial genome in these species. Finally, we outline some possible future research directions to identify the factors influencing mitochondrial genome evolution in this group.

Metazoans have relatively conserved mitochondrial gene content and structure. These organellar genomes in animals usually contain a set of 13 protein-coding genes, two ribosomal RNA genes, and approximately 22 tRNA genes. They typically occur in the form of a circular molecule approximately 15–20 kb in size (Boore 1999; Gissi *et al.* 2008). Nevertheless, some notable exceptions exist, such as a lack of the *atp8* gene in many nematodes (Hu and Gasser 2006). Exceptions to overall structure are seen in the rotifer *Brachionus plicatilis*, whose mitochondrial genome consists of two separate circular DNA molecules (Suga *et al.* 2008), whereas those of many cnidarians consist of one or more linear molecules, which have also lost most of their tRNA genes (Kayal *et al.* 2012).

Isopods represent another important deviation from the typical metazoan mitochondrial genome architecture. In many species, the mitochondrial genome has a unique structure consisting of both linear molecules approximately 14 kb in length, as well as circular molecules composed of two of the linear molecules joined in a head-to-head arrangement (Raimond *et al.* 1999; Marcadé *et al.* 2007). Mapping this trait onto the isopod phylogeny reveals that it has either evolved independently multiple times or evolved in an early isopod ancestor but was subsequently lost in some lineages (Doublet *et al.* 2012). The relative gene order within the linear monomeric unit, however, is conserved in most of the isopods that have been sequenced so far, at least for protein-coding and ribosomal RNA genes (Kilpert and Podsiadlowski 2006; Marcadé *et al.* 2007; Kilpert *et al.* 2012).

The mechanisms of mitochondrial DNA replication and transmission in isopods with this dual linear/circular structure are not yet fully known. However, inverted repeat sequences forming telomeric hairpins at the ends of the monomer sequence may facilitate conversion between the two forms of the mitochondrial DNA, and may also stabilize the linear molecules or aid in their replication (Doublet *et al.* 2013). The monomer sequences within an individual show almost no sequence divergence, suggesting that gene conversion may maintain sequence homogeneity (Marcadé *et al.* 2007). One important exception to this observation, however, is the presence of a stably transmitted heteroplasmic site. This heteroplasmic site alters the anticodon in a tRNA gene, allowing this single locus to code for both tRNA^Ala^ and tRNA^Val^ (Marcadé *et al.* 2007). Remarkably, this heteroplasmic site is shared across a number of species that last shared a common ancestor at least 30 million years ago (Doublet *et al.* 2008), suggesting strong selective pressure to maintain it.

Here, we further explore the evolution of mitochondrial genomes in terrestrial isopods with complete mitochondrial genome sequences for two additional species, *Trachelipus rathkei* and *Cylisticus convexus*. The relative order of protein-coding and rRNA genes is conserved, whereas the arrangement of tRNA genes is more variable. Strikingly, we found two additional heteroplasmic sites that also alter anticodon sequences in tRNA genes and are both conserved across these two species. We discuss the implications of these findings for the evolution of tRNA genes and mitochondrial genome architecture in general.

## Materials and Methods

### Sequencing and assembly of the *T. rathkei* mitochondrial genome

Wild-caught specimens of the terrestrial isopod *Trachelipus rathkei* were obtained from Rice Creek Field Station in Oswego, New York, USA. Genomic DNA was extracted from ventral muscle/nerve, leg, and gonadal tissue from one male and one female, using a DNeasy Blood and Tissue DNA extraction kit (Qiagen). The two samples were barcoded, pooled, and sequenced in a single 2×100 lane on an Illumina HiSeq 2000 at the University at Buffalo, providing a total of ∼140 million sequence read pairs.

Briefly, adapter sequences were removed and quality filtering was performed using Trimmomatic (Bolger *et al.* 2014). An initial assembly of all sequence reads was performed using Minia 1.6906 (Chikhi and Rizk 2012; Salikhov and Sacomoto 2013). Putative mitochondrial sequences from this assembly were identified by looking for contigs with relatively high coverage, estimated by mapping reads using bwa (Li and Durbin 2009; Li 2013) and obtaining depth using samtools (Li *et al.* 2009), and showing high similarity to the *Armadillidium vulgare* mitochondrial genome (Marcadé *et al.* 2007) by BLAST searches (Altschul *et al.* 1990). This search identified a single contig of approximately 13 kb in length with ∼1600× and 680× coverage in the male and female samples, respectively.

To obtain a more complete mitochondrial genome assembly, we used an iterative mapping approach, similar to those adopted by others (Hahn *et al.* 2013), using a custom script. All reads mapping to the putative mitochondrial contig, along with their mates, were re-assembled. Reads mapping to the new assembly were then identified, and this cycle was repeated for 10 iterations. Finally, a separate assembly was constructed for each of the two sequenced individuals, across a range of parameter values. We selected the assembly with the single longest contig as our final reference mitochondrial genome for *T. rathkei*. For full details of all assembly procedures, please see the Supporting Information, File S1.

### Sequencing and assembly of the *C. convexus* mitochondrial genome

The *C. convexus* mitochondrial genome was assembled from data generated as part of the sequencing of the *Wolbachia* endosymbiont of *C. convexus* (M. Badawi, B. Moumen, P. Grève, and R. Cordaux, unpublished data). Briefly, gonads of 30 *C. convexus* female individuals (all sisters), from our laboratory line CCw (derived from individuals caught in Avanton, France, in 1993) were pooled, and total genomic DNA extraction was performed using a standard phenol-chlorophorm protocol. The sample was sequenced by GenoScreen (Lille, France) on 1/4 and 1/8 runs of a 454 GS FLX sequencer using the Titanium chemistry, providing a total of ∼110 Mb of sequence data.

Read quality was checked with FastQC and reads were assembled *de novo* (including quality filtering and trimming) using the gsAssemler software implemented in Newbler version 2.6, with default parameters, except a seed step of 1 was used to improve sensitivity. All contigs were mapped against the *A. vulgare* mitochondrial genome (Marcadé *et al.* 2007) using MAUVE version 2.3 (Darling *et al.* 2010) to identify putative mitochondrial contigs. The resulting contigs were then manually resolved into a single contig using the de Bruijn graph of contigs. This contig was approximately 14 kb in length with ∼450× coverage.

### Annotation

The *T. rathkei* mitochondrial genome was initially annotated using the MITOS web server (Bernt *et al.* 2013), which identifies protein-coding and rRNA genes using homology searches, and a variant of the MITFI algorithm (Juhling *et al.* 2012) to identify putative tRNA genes. We used the invertebrate mitochondrial genetic code, and the default settings for all other parameters. We also used ARWEN (Laslett and Canback 2008) to provide a complementary set of predicted tRNA genes. Finally, the annotations for the protein-coding and rRNA were manually corrected, aided by BLAST searches against other isopod mitochondrial genes, because in many cases the initial sequences generated by the automated software lacked start or stop codons or were obviously incomplete (*e.g.*, rRNA genes were too short). The *C. convexus* mitochondrial genome was annotated similarly, using BLAST searches to identify protein-coding and ribosomal RNA genes, and MITFI and ARWEN to identify tRNA genes.

### Sequence polymorphisms

For *T. rathkei*, the putative mitochondrial reads from each individual were mapped to the reference mitochondrial genome generated above using bwa. For *C. convexus*, all sequencing reads were mapped to the reference mitochondrial genome generated above using Bowtie2 (Langmead and Salzberg 2012). Full pileup files were generated for each species using the samtools pileup command. All positions with at least 100× coverage in which a nonreference allele was present at a frequency greater than 0.2 were identified as putative polymorphisms.

Selected polymorphisms were confirmed by Sanger dye-terminator sequencing of the DNA samples used for high-throughput sequencing, as well as two *T. rathkei* individuals caught in Oswego (United States) and 12 *C. convexus* individuals originating from various geographic locations (Avanton and Villedaigne in France, and North Carolina in the United States).

## Results

### Assembly, annotation, and gene order

**Figure 1:**
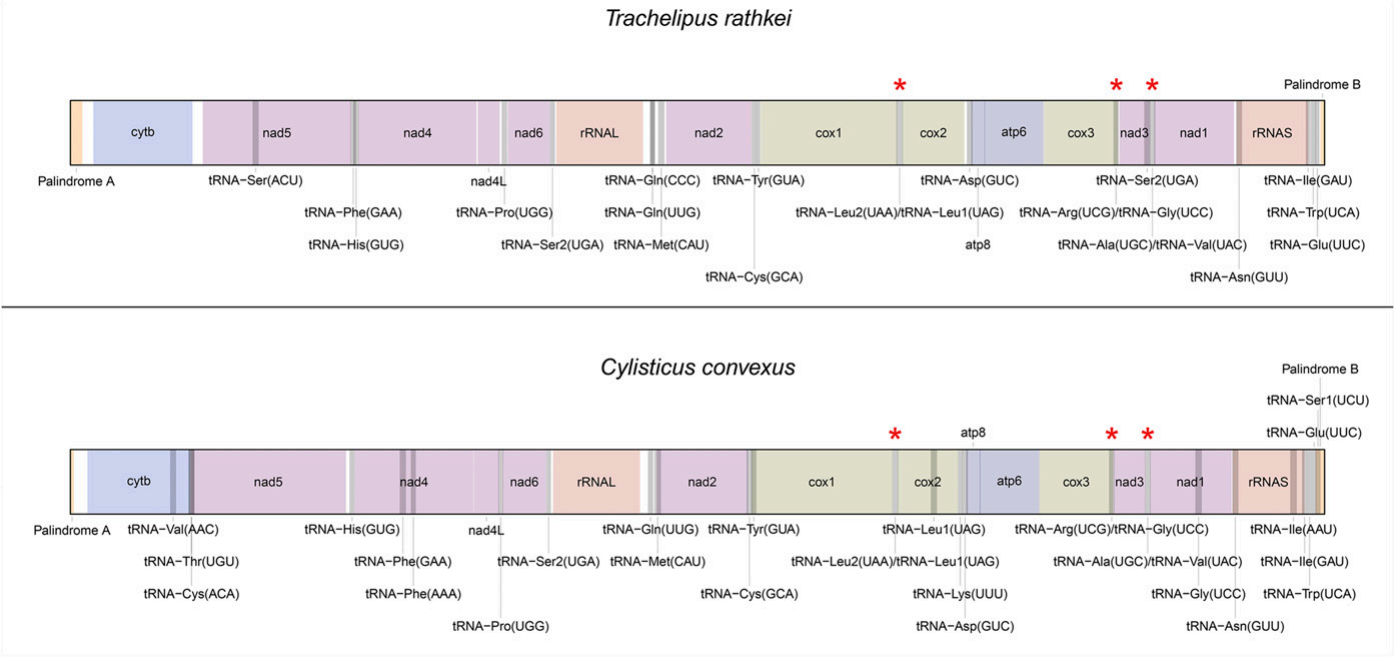
Organization of the monomeric unit of the mitochondrial genomes of *Trachelipus rathkei* and *Cylisticus convexus*. Asterisks represent the location of conserved heteroplasmic sites.

The mitochondrial genome assemblies of *T. rathkei* and *C. convexus* were 14,129 and 14,154 bp long, respectively. Despite their atypical structure that has generally hampered previous efforts to obtain complete mitochondrial genomes of terrestrial isopods (Doublet *et al.* 2013), next-generation sequencing allowed us to recover the complete mitochondrial genomes of *T. rathkei* and *C. convexus*. This conclusion is supported by the fact that both mitochondrial genomes have palindromic structures at their termini that may form telomeric hairpin structures, as expected in linear mitochondrial genomes (Doublet *et al.* 2013) (Figure 1).

Gene order for protein-coding and rRNA genes was conserved between these two species and also identical to that of *A. vulgare* and other terrestrial isopods (Marcadé *et al.* 2007; Kilpert *et al.* 2012) (Figure 1). The positioning of putative tRNA genes, however, was more variable between the two species (Figure 1). As a whole, the mitochondrial genomes of the two species displayed ∼72% sequence identity when aligned by ClustalW2 (Larkin *et al.* 2007). The assembled sequences and annotations have been deposited in GenBank under accession numbers KR013001 and KR013002.

### Heteroplasmic sites in *T. rathkei*

**Figure 2:**
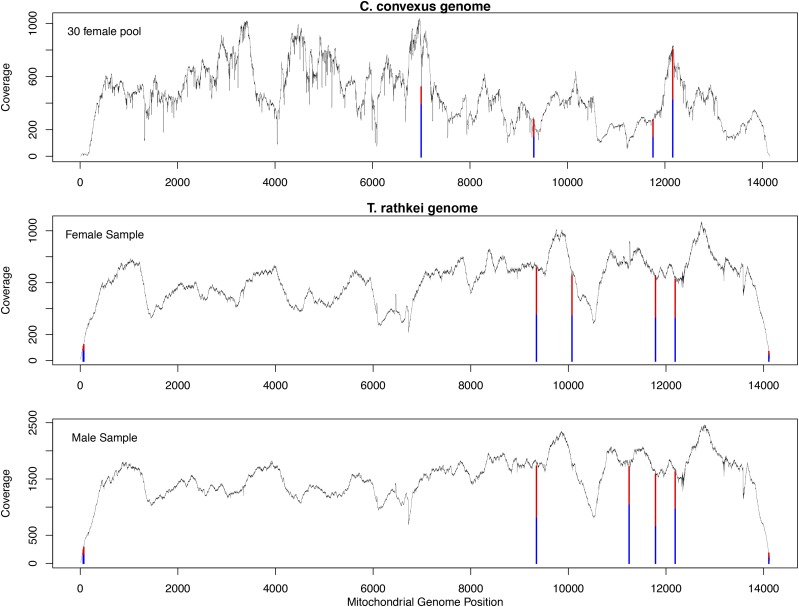
Sequencing depth across the monomeric unit of the mitochondrial genomes of *Cylisticus convexus* and each of the two *Trachelipus rathkei* samples. Vertical bars represent putative heteroplasmic sites detected by mapping reads to the assembled genomes. The blue portion of each bar represents the frequency of the reference allele in the sequencing reads, whereas the red portion represents the frequency of the alternate allele.

Coverage across the *T. rathkei* mitochondrial genome was quite high, >300× across most of the genome in each sample, except at the very ends of the monomer sequence (Figure 2). There were no fixed sequence differences between the two individuals sequenced. However, eight heteroplasmic sites were identified (Figure 2, Table 1). Six of these heteroplasmic sites were detected with confidence in both individuals, and each allele occurred at a frequency of approximately 0.5 in all of these cases. Three of the six occurred toward the ends of the assembled monomer sequence (positions 62, 73, and 14107), and thus had relatively low coverage. The remaining three occurred in putative tRNA genes, and in the anticodon of each (positions 9346, 11785, and 12188). These three were supported by high coverage in both individuals; we also confirmed all three in additional unrelated wild-caught individuals using Sanger dye-terminator sequencing (Figure S1).

**Table 1:** Mitochondrial sequence heteroplasmies in *Trachelipus rathkei*

Pos.	Ref. Allele	Alt. Allele	Alt. Freq., M	Cov., M	Alt. Freq., F	Cov., F	Notes
62	T	A	0.45	252	0.39	111	Noncoding palindrome sequence
73	T	A	0.47	299	0.39	127	Noncoding palindrome sequence
9346*	G	A	0.53	1740	0.51	718	tRNA Leu1/Leu2 anticodon shared with *C. convexus*
10074	G	A	0	1737	0.47	657	Noncoding
11244	A	—	0.39	1726	0.04	756	*cox3* frameshift, affecting amino acids 92 – 277; creates premature stop codon at position 106
11785*	G	C	0.58	1594	0.49	651	tRNA Arg/Gly anticodon shared with *C. convexus*; also causes *cox3* substitution (E273Q)
12188*	A	G	0.41	1638	0.47	629	tRNA Ala/Val anticodon shared with *C. convexus*
14107	T	A	0.5	193	0.46	74	Noncoding palindrome sequence

Alt. freq., Cov. M, and Cov. F indicate the frequency of the alternative allele in the sequence reads, and the total coverage at that site, in the male and female samples, respectively. The — at site 11244 indicates that the alternate allele is a 1-bp deletion. *Heteroplasmic sites that were confirmed by Sanger sequencing.

Finally, there were two additional putative heteroplasmic sites present at high frequencies in only one of the two individuals. One of these, unique to the female individual, was in a noncoding region (position 10074). The other was not a substitution, but a single base-pair deletion causing a frame shift within the *cox3* gene (position 11244). Interestingly, the alternate allele at this position was detected in the female sample, but at a very low frequency (4%).

We were unable to identify the haplotype phase for most pairs of heteroplasmic sites, because they were too distantly spaced to be covered by single reads or read pairs in the Illumina data. However, for the polymorphisms at positions 62 and 73, we were able to identify reads covering both sites. In all cases, reads contained either both reference alleles or both alternate alleles (male sample: 133 reads with 62A and 73A, 119 reads with 62T and 73T; female sample: 68 reads with 62A and 73A, 40 reads with 62T and 73T; Figure S2).

### Heteroplasmic sites in *C. convexus*

Coverage across the *C. convexus* mitochondrial genome was 450× on average (Figure 2). A total of four potential heteroplasmic sites were identified in *C. convexus* (Figure 2, Table 2). One site was an A/T polymorphism located at the boundary between A_2_ and T_8_ homopolymers (position 6998), which are known to be error-prone in 454 sequencing. Therefore, this putative heteroplasmy most likely reflects sequencing/mapping errors or artifacts.

**Table 2:** Mitochondrial sequence heteroplasmies in *Cylisticus convexus*

Position	Reference Allele	Alternative Allele	Alt. Frequency	Coverage	Notes
6998	T	A	0.25	526	*nad2* substitution (F118I) located at boundary between A_2_ and T_8_ homopolymers
9309*	A	G	0.46	277	tRNA Leu1/Leu2 anticodon shared with *T. rathkei*
11755*	G	C	0.47	273	tRNA Arg/Gly anticodon shared with *T. rathkei*; also causes *cox3* substitution (D274H)
12160*	G	A	0.47	806	tRNA Ala/Val anticodon shared with *T. rathkei*

Alt. frequency, frequency of the alternative allele in the sequence reads. *Heteroplasmic sites that were confirmed by Sanger sequencing.

The other three heteroplasmic sites occurred in the anticodon of putative tRNA genes (positions 9309, 11755, and 12160), and each allele occurred at a frequency of approximately 0.5 in all three cases. Interestingly, they appear to be orthologous to the three confirmed heteroplasmic sites in *T. rathkei* tRNA anticodons. The heteroplasmy at position 12160 has previously been reported to be widespread among terrestrial isopods, including *C. convexus* (Doublet *et al.* 2008). The heteroplasmies at positions 9309 and 11755 were confirmed by Sanger sequencing with the original DNA pool used for 454 sequencing, as well as a set of 12 individual samples originating from diverse geographic locations (Figure S3).

The heteroplasmy at position 9309 is >2 kb away from the other heteroplasmies, which precluded identification of haplotype phase. By contrast, the heteroplasmies at positions 11755 and 12160 are ∼400 bp away from each other, which falls in the range of 454 sequencing read length, thereby enabling identification of haplotype phase. Consistently, we identified 35 reads covering both sites: 18 contained both reference alleles (11755G and 12160G) and 17 contained both alternate alleles (11755C and 12160A) (Figure S4).

## Discussion

We have successfully assembled ∼14.1 kb of mitochondrial genome sequences for two additional species of terrestrial isopods, *Trachelipus rathkei* and *Cylisticus convexus*, using second-generation sequencing approaches. Two lines of evidence suggest that these assemblies are complete: (i) the termini of the mitochondrial monomer sequence in *A. vulgare* possess telomeric hairpin sequences, and these features are present in both of our assemblies; and (ii) these assemblies are both longer than the complete mitochondrial sequence in *A. vulgare* (13939 bp) (Doublet *et al.* 2013). Moreover, we find the full set of protein-coding and ribosomal RNA genes typically found in animal mitochondrial genomes. The relative ordering of these genes is identical between the two species (Figure 1). This is not surprising given that mitochondrial gene order is also conserved across most other isopods that have been studied, with a few exceptions (Kilpert *et al.* 2012).

Despite conservation in the arrangement of protein-coding and ribosomal RNA genes, the tRNA genes provide some surprising evolutionary insights. Most notably, we have discovered multiple heteroplasmic sites in tRNA genes that are also common to both species (Table 1, Table 2, Figure 2). Although one of these sites was previously known (Marcadé *et al.* 2007; Doublet *et al.* 2008), two are novel. The fact that all three of these shared heteroplasmies alter anticodon sequences, allowing these loci to function as dual tRNA genes, seems unlikely to be a chance occurrence. Although it is unclear exactly how long ago *T. rathkei* and *C. convexus* diverged, they are classified in different families, and the relatively low sequence identity of their mitochondrial genomes suggests they shared a common ancestor at least millions of years ago. Strong selection therefore seems likely to have maintained the crucial dual function of these genes for a very long time, and the atypical organization of the mitochondrial genome probably facilitates the evolution of such stable heteroplasmic sites. Conversely, selection to maintain the heteroplasmic sites probably also maintains the unusual linear/circular architecture of these mitochondrial genomes.

In contrast to the conserved heteroplasmic tRNA loci, evolutionary turnovers in the locations of other tRNA genes appear to be more common. One possible explanation for this apparent contradiction is that tRNA genes are normally evolutionarily labile, but that the dual heteroplasmic state represents a sort of evolutionary “trap.” However, we also recognize that the annotations themselves may be imperfect or incomplete. For instance, some tRNA genes were only detected by one of the two software packages we used, and some tRNA genes appear to be missing altogether (*e.g.*, tRNA^Thr^ gene in *T. rathkei*). While it is possible that these tRNA genes are truly absent from these mitochondrial genomes, we cannot rule out the alternative possibility that they were simply missed by the annotation software. tRNA genes with unusual secondary structures, or subject to substantial RNA editing, for instance, might be especially difficult to detect; some of the identified genes lack the D and/or T arm (Figure S5). Although this is not especially unusual—mitochondrial tRNA genes missing arms have been noted in other lineages (*e.g.*, Masta and Boore 2008), and editing of tRNA molecules is suspected in the isopod *Ligia oceanica* (Kilpert and Podsiadlowski 2006)—it is still possible that additional tRNA genes with reduced structures were present but not successfully identified.

In any case, these were not the only heteroplasmic sites detected. In *C. convexus*, one additional polymorphism was found (site 6998) but is probably an artifact because it occurs in a homopolymer sequence, which are known to be error-prone in 454 data. In *T. rathkei*, we found evidence of three others in the terminal palindromes at the ends of the mitochondrial genome (sites 62, 73, and 14107). Two additional heteroplasmic sites in *T. rathkei* (10074 and 11244) were polymorphic, found in only one of the two individuals we sequenced. One of these sites (10074) was in one of the few apparently nonfunctional areas of the mitochondrial genome, whereas the other (11244) was a frameshift-causing 1-bp deletion in the *cytochrome b* gene, almost certainly resulting in a nonfunctional product. Although such a mutation in an essential mitochondrial gene would normally be expected to be deleterious, the presence of another functional copy of the gene likely mitigates those effects. Interestingly, the loss-of-function allele was also detected at a very low frequency of 4% in the other individual we sequenced. Although this low-frequency heteroplasmy might be explained by sequencing or alignment artifacts, an intriguing alternative possibility is that gene conversion coupled with selection may be acting at this site to maintain a functional *cytochrome b* sequence.

The relative rarity of fixed heteroplasmic sites in these mitochondrial genomes (3–6 sites out of ∼14 kb) suggests that gene conversion is a potent homogenizing force in isopod mitochondrial genomes. Concerted evolution has also been observed in duplicated regions in mitochondrial genomes in other lineages (Ogoh and Ohmiya 2007; Tatarenkov and Avise 2007). However, a high frequency of gene conversion would raise the question of how heteroplasmic tRNA genes are maintained over such long time scales. Of course, as mentioned, selection is likely to play a role. We also cannot discount the possibility that these sites somehow escape gene conversion, for example, if they play a mechanistic role in the gene conversion process. Another possibility is that they may be secondarily edited after gene conversion events. These hypotheses are not mutually exclusive, and could even act together.

Further study is clearly needed on the processes influencing mitochondrial variation and evolution in this lineage, including on the mechanisms underlying gene conversion and recombination, on mutation rates and the frequency of gene conversion events, and on the strength of selection on different types of variants. For instance, further haplotype phasing within individuals could shed light on the tempo of gene conversion; the presence of all possible haplotypes within each individual would suggest rapid and dynamic recombination of mitochondrial monomer units within individuals. Preliminarily, there appear to be just two distinct haplotypes across two closely linked heteroplasmic sites near one end of the *T. rathkei* mitochondrial DNA sequence (Figure S2), but haplotype phasing at the other sites was impossible with our short-read data, even when taking paired read information into account. Similarly, in *C. convexus*, just two haplotypes are found in equal numbers across the heteroplasmies at sites 11755 and 12160 (Figure S4). Studying such sites across multiple individuals in a family would answer important questions about mechanisms of transmission. For example, while the tRNA^Ala^/tRNA^Val^ heteroplasmy was stably transmitted in one study in *A. vulgare* (Doublet *et al.* 2008), repeating such studies in families with multiple heteroplasmic sites, especially combined with haplotype phase information, would confirm pure maternal cytoplasmic inheritance and stable transmission of multiple heteroplasmic sites. Finally, examining many individuals across multiple polymorphic populations, combined with modeling studies, would shed light on the microevolutionary dynamics of these sites (*e.g.*, frequency of gene conversion and strength of selection). Plummeting sequencing costs, combined with increasing throughput and read lengths, should make sequencing whole mitochondrial genomes in many individuals feasible, providing rich opportunities to answer these questions in the future.
